# Sustained suppression of IL-18 by employing a vaccine ameliorates intestinal inflammation in TNBS-induced murine colitis

**DOI:** 10.2144/fsoa-2018-0125

**Published:** 2019-07-30

**Authors:** Qingdong Guan, Richard Warrington, Sem Moreno, Gefei Qing, Carolyn Weiss, Zhikang Peng

**Affiliations:** 1 Department of Immunology, University of Manitoba, Winnipeg R3E 3P4, Canada; 2 Department of Pediatrics & Child Health, University of Manitoba, Winnipeg, R3E 3P4, Canada; 3 Department of Internal Medicine, University of Manitoba, Winnipeg, R3E 3P4, Canada; 4 Cellular Therapy Laboratory, CancerCare Manitoba, Winnipeg, R3A 1R9, Canada; 5 Department of Pathology, University of Manitoba, Winnipeg, R3E 3P4, Canada

**Keywords:** IL-18, immunotherapy, murine colitis, peptide-based vaccine

## Abstract

**Aim:**

To develop IL-18 peptide-based virus-like particle vaccines that elicit autoantibodies against IL-18 and to evaluate the *in vivo* effects of the vaccines in murine colitis.

**Methods:**

Recombinant IL-18 vaccines were constructed, and the effects of the vaccines were evaluated in trinitrobenzene sulfonic acid-induced acute and chronic colitis in mice.

**Results:**

Two murine IL-18 peptide-based vaccines (A and D) were developed, which induced relative long-lasting specific antibodies against IL-18. Vaccine-immunized mouse antisera could partially block IL-18-induced IFN-γ production *in vitro*. Mice receiving vaccine D, not vaccine A, had a significant decrease in intestinal inflammation, collagen deposition and pro-inflammatory cytokine levels in colon tissue.

**Conclusion:**

IL-18 vaccine may provide a potential therapeutic approach in the treatment of Crohn’s disease.

## Lay abstract

Many proinflammatory cytokines, including IL-18, play important roles in exaggerating the disease progression of inflammatory bowel disease (IBD). Inflammatory bowel disease is a chronic autoimmune disease, which usually requires long-term treatment. Blocking these proinflammatory cytokines by using monoclonal antibodies has shown certain clinical efficacy, but it requires repeated injection of these antibodies. To induce relative long-lasting antibodies, we developed IL-18 peptide-based virus-like particle vaccines and evaluated their therapeutic effects in a murine colitis model. Our results showed that immunization of mouse with IL-18 peptide-based vaccine could improve murine colitis, which indicated this vaccine strategy might be a potential treatment approach.

It is widely accepted that Crohn’s disease is caused by an overly aggressive type 1 helper T cells (Th1) immune response and excessive IL-23/Th17 pathway activation to bacterial antigens in genetically predisposed individuals [1–3]. IL-18 is considered as a Th1 cytokine of the IL-1 family, produced by cells of monocytic lineage, dendritic cells and intestinal epithelial cells [4,5]. IL-18 promotes the proliferation of Th1 lymphocytes and the production of IFN-γ and tumor necrosis factor (TNF). Therefore, it is associated with a number of diseases such as arthritis and myocardial hypertrophy [5]. The pro-inflammatory role of IL-18 in inflammatory bowel disease (IBD) has been supported by studies in patients with IBD. IL-18 levels in sera and colon mucosal biopsies are significantly elevated in patients with Crohn’s disease, and correlate with disease activity and inflammatory markers [6,7]. In animal studies, IL-18 levels are significantly increased in experimental colitis. Blocking IL-18 with its monoclonal Ab or binding protein or the knocking out of IL-18 results in a dramatic attenuation of intestinal inflammation [8,9]. In addition to that, polymorphisms in the *IL18R1–IL18RAP* locus are found to be associated with the susceptibility of Crohn’s Disease [10,11], and the gene *IL-1R2* (containing IL-18RPAP) has been recently identified as one of the ulcerative colitis risk factors [12]. These studies indicate that IL-18 may be a therapeutic target in the treatment of IBD [8].

Currently used human monoclonal antibodies that target cytokines, such as infliximab, have disadvantages of a short half-life (infliximab is 9.5 days) and the development of antibodies to the infused monoclonal antibodies [13]. To overcome these disadvantages, vaccines against overexpressed endogenous cytokines have emerged as a potential new biotherapy that may offer long-term efficacy with fewer adverse effects [14–16]. Our laboratory has successfully designed cytokine vaccines by inserting a small peptide derived from the target cytokine into a carrier protein, hepatitis B core antigen (HBcAg), using molecular engineering methods. This type of vaccine presents as virus-like particles and elicits sufficient autoantibodies to the target cytokine without the use of an adjuvant and result in the amelioration of the disease [15,16].

In the present study, for the first time, we developed IL-18 peptide-based virus-like particle vaccines and evaluated the effects of these vaccines in acute and chronic murine colitis.

## Methods

### Animals

Female BALB/c mice (7–8 weeks old) purchased from Charles River Laboratories (QC, Canada) were maintained at Central Animal Care Services, University of Manitoba. All protocols used were approved by the University Animal Ethics Committee.

### Preparation & identification of vaccines & carrier HBcAg

Antigenic peptide prediction was performed based on the occurrence of amino acid residues in experimentally known segmental epitopes (http://bio.dfci.harvard.edu/Tools/antigenic.html) and the DNAstar software. Six vaccines with different peptides (Table 1) were developed as previously described [15,17]. Briefly, using the vector pThio-His, a plasmid containing either: ‘vaccine’ - HBcAg inserted with one of the six chosen peptides or ‘carrier’ – truncated HBcAg (amino acids 1–149) was transformed into *Escherichia coli* DH_5α_ cells. The recombinant plasmids were then identified by restriction endonucleases digestion and SDS-PAGE. Expression of the vaccine or carrier was induced. They were purified by a combination procedure consisting of ultrasonication lysis, ammonium sulfate precipitation and size exclusion chromatography with Sepharose CL-4B (Sigma-Aldrich, ON, Canada). Endotoxin in the recombinant proteins was removed with Affi-prep Polymyxin Matrix (Bio-Rad, ON, Canada). To determine their antigenicity, mice were immunized with each vaccine or the carrier protein three-times (first dose 100 μg/200 μl, second dose 50 μg/200 μl and third dose 25 μg/200 μl) at a 2-week interval (n = 4 mice/group). A total of 5 weeks later, mice were immunized with 25 μg/200 μl again. Sera were collected at indicated times to detect IL-18-specific IgG levels by ELISA.

**Table 1.** Selected antigenic peptides from mouse IL-18 and resulting recombinant peptide-based vaccines.

Peptide no.	Amino acid sequence	Soluble protein (vaccine)
A	^23^DKRQPVFED^31^	Yes
B	^37^QSASEPQT^44^	Yes
C	^51^YKDSEVRGL^59^	No
D	^84^EMDPPENIDDIQS^96^	Yes
E	^125^CQKEDDAF^132^	No
F	^137^KKKDENGDKS^146^	No

### Measurements of antibodies & cytokines by ELISA

Serum IL-18-specific IgG levels were assayed by using ELISA techniques established in our laboratory [17,18], in which, IL-18 protein (0.25 μg/ml) (PeproTech, NJ, USA) was coated on a microplate followed by incubation with diluted test serum from individual mouse (1:200 dilution). ALP-conjugated rabbit anti-mouse IgG (PeproTech) was used as the secondary antibody, followed by addition of a substrate for developing a color reaction. The results were expressed using optical density at 405 nm (OD_405_).

Due to the limited quantity, the serum from the same group was pooled to evaluate the antibody titre and *in vitro* inhibition assay. Serum-specific IgG titers were assayed by ELISA using pooled sera from each group and the results were expressed using ‘titer’, the reciprocal of the highest dilution in which the OD_405_ was 0.2, twice that of the corresponding control sera when its OD_405_ was 0.10.

To measure the cytokine levels in the colon tissue, frozen colonic samples from individual mouse were mechanically homogenized in buffer containing 1 M Tris-HCl, 3 M NaCl and 10% Triton supplemented with protease cocktail (Sigma-Aldrich). Samples were then frozen (-70°C) and thawed (37°C) three-times, followed by centrifugation at 14,000 rpm. for 30 min at 4°C. Supernatants were frozen at -70°C until assay. Cytokine concentrations (IFN-γ, TNF and IL-18) in the supernatants of colon tissues were measured by ELISA techniques established in our laboratory according to manufacturer’s instructions [16,17], in which, primary anti-cytokine antibody (1 μg/ml, BD Bioscience, CA, USA) was coated on a microplate followed by incubation with testing supernatants obtained from individual mouse or with cytokine standards. Biotinylated anti-cytokine detection antibody (1 μg/ml, BD Bioscience) was added and incubated, then followed by incubation with Avidin-horseradish peroxidase, and finally, the substrate was added to develop the color reaction.

### Protocols for induction of chronic colitis & vaccine immunization

The vaccine was first *in vivo* evaluated in a 2,4,6-trinitrobenzene sulfonic acid (TNBS)-induced acute colitis in which mice were subcutaneously injected three-times at a 2-week interval with vaccine, vaccine carrier HBcAg or saline (first dose 100 μg/200 μl, second dose 50 μg/200 μl and third dose 25 μg/200 μl). A total of 2 weeks later, mice were intrarectally challenged with TNBS (Sigma-Aldrich) twice (1.5 and 2.0 mg, respectively) at a 1-week interval to induce acute colitis (Figure 2A) as we described previously [19]. Mice were sacrificed 1 week after the second TNBS challenge. Colons and blood samples were collected and processed according to different assays.

The vaccine was further evaluated in TNBS-induced chronic colitis, in which mice were immunized four-times with vaccine or carrier (first dose 100 μg/200 μl, second dose 50 μg/200 μl, third dose 25 μg/200 μl and fourth dose 25 μg/200 μl) or saline (n = 10). 2 weeks later after the third vaccination, chronic colitis was induced by seven weekly administrations of increasing doses of TNBS (1.0–2.5 mg; Figure 2A). 1 week later after the last TNBS delivery, mice were sacrificed. Colons and blood samples were collected and processed.

To test serum IL-18-specific antibody responses, serum samples were collected at weeks 3, 5, 8, 10 and 13.

### Histological examination

Ten percent buffered formalin-fixed and paraffin-embedded colon sections were cut and stained with haemotoxylin and eosin (H&E) for the evaluation of intestinal inflammation. Histological scoring was evaluated by a pathologist blinded to the source of treatment, based on the method previously described [20]. During each histological examination, three different parameters were used: severity of inflammation, depth of injury and crypt damage. All values were added to a sum, in which the maximum possible score was 10.

### Soluble collagen assay

Colons were homogenized in 0.5 M acetic acid containing 1 mg of pepsin (at a concentration of 10 mg of tissue/5 ml of acetic acid solution). The resulting mixture was then incubated and stirred for 24 h at 4°C. Total soluble collagen content of the mixture from an individual mouse was determined with a Sircol Collagen Assay Kit (Biocolor, County Antrim, UK) [21]. Acid soluble type I collagen supplied with the kit was used to generate a standard curve.

### Measurement of *in vitro* bioactivity of vaccine-induced IL-18-specific IgG

To examine whether vaccine-induced sera could inhibit the biological activity of IL-18-induced production of IFN-γ, 2 × 10^5^ spleen cells of BALB/c mice in triplicate wells were stimulated with anti-murine CD3ε/CD28 (2 μg/ml) in the presence of recombinant murine IL-12 (2 ng/ml) and IL-18 (10 ng/ml) protein, with the total volume 200 μl per well for 4 days in 96-well plate. Different dilutions of a pooled serum obtained from the same vaccine-immunized mice or a pooled serum from carrier-immunized mice were added into the culture. After 96 h, supernatants were collected to detect the expression levels of IFN-γ by ELISA. The inhibition percentage of mouse antiserum was calculated as follows:

$$\text{Inhibition}\;(\%)=\frac{\text{IFN}-\gamma \;\text{of}\;\text{carrier}\;\text{serum}\;-\;\text{IFN}-\gamma \;\text{of}\;\text{test}\;\text{serum}}{\text{IFN}-\gamma \;\text{of}\;\text{carrier}\;\text{serum}}100\%$$

### Statistical analyses

Values were expressed as mean ± SD. Differences between experimental groups were assessed by ANOVA followed by Newman–Keuls multiple comparison test (GraphPad Prism, CA, USA). The p-value < 0.05 was considered statistically.

## Results

### Immunization with IL-18 peptide-based vaccines induces relative long-lasting antibodies against IL-18

Six peptides were selected from mouse IL-18 based on the antigenic index, flexibility, surface probability and hydrophilicity (Table 1). To break self-tolerance, truncated hepatitis B core antigen (HBcAg) was used as a vaccine carrier. Each peptide was inserted into the vector plasmid pThio-HBcAg using the methods described previously [17]. Six recombinant vaccine proteins were expressed using *E. coli* and purified appropriately. Based on the formation of virus-like particles, three IL-18 peptide-vaccines, named vaccine A, B and D, were obtained for further characterization.

**Figure 1.**
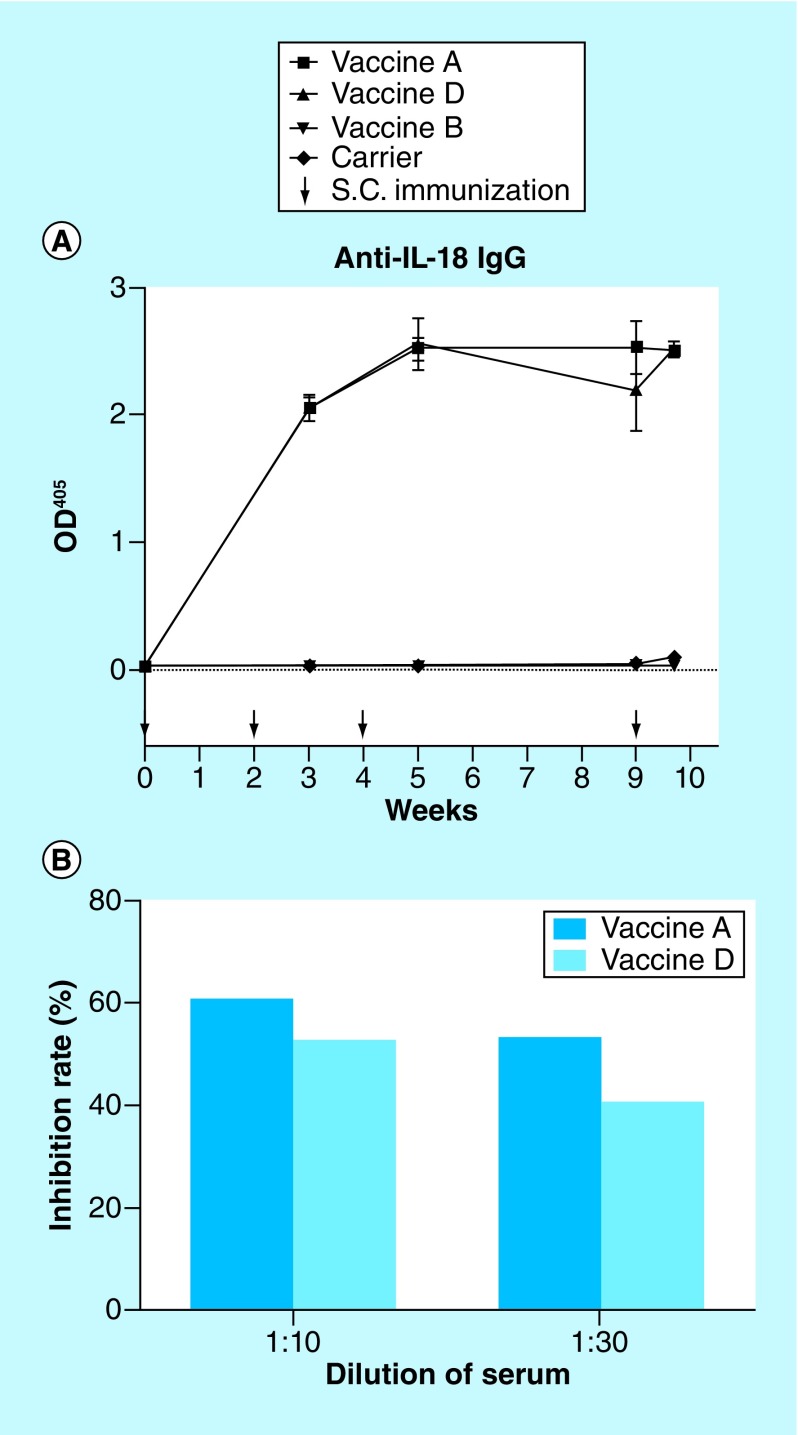
IL-18-specific IgG antibody responses induced by three IL-18 peptide-vaccines and *in vitro* inhibition tests. Female BALB/c mice (n = 4/group) were subcutaneously immunized three-times (first dose 100 μg/200 μl, second dose 50 μg/200 μl and third dose 25 μg/200 μl) of each vaccine (vaccines A, B and D), carrier or saline at a 2-week interval. A total of 5 weeks later, the mice were boost-immunized with 25 μg/200 μl again. Sera were obtained from the individual mouse at the indicated weeks and diluted 1/200 for determination of specific IgG levels by ELISA. **(A)** Serum IL-18-specific IgG levels. **(B)** The *in vitro* inhibition of anti-IL-18 induced by vaccines was evaluated through inhibiting IL-18-induced IFN-γ secretion from splenocytes.

To determine the antigenicity, mice were immunized with each vaccine or the carrier four-times (Figure 1A). Sera were collected and tested for IL-18-specific IgG response by ELISA. As shown in Figure 1A, vaccines A and D induced significantly high levels of IL-18-specific IgG antibodies, while mice receiving vaccine B or carrier had no detectable specific antibodies. The IL-18 antibodies induced by vaccines A and D remained high levels and lasted for over 1 month, similar to our previous reports on an IL-12/IL-23p40 peptide-based vaccine [19]. The titers of anti-IL-18 IgG were up to 180,000 for vaccine A and 120,000 for vaccine D.

### Anti-IL-18 induced by IL-18 peptide-based vaccines partially block IL-18-induced IFN-γ secretion *in vitro*

Next, we evaluated the inhibition effects of antibody induced by IL-18 peptide-based vaccines. *In vitro* inhibition tests showed antisera from mice immunized with vaccine A or D-inhibited IL-18-induced IFN-γ secretion of splenocytes in a dose-dependent manner, indicating that vaccine-induced IL-18-specific antibodies were able to inhibit the biological function of IL-18 *in vitro*. Compared with vaccine D, vaccine A had a better ability to induce specific antibodies and block the biological functions of IL-18.

### Immunization with IL-18 peptide-based vaccine D ameliorate TNBS-induced acute & chronic colitis

**Figure 2.**
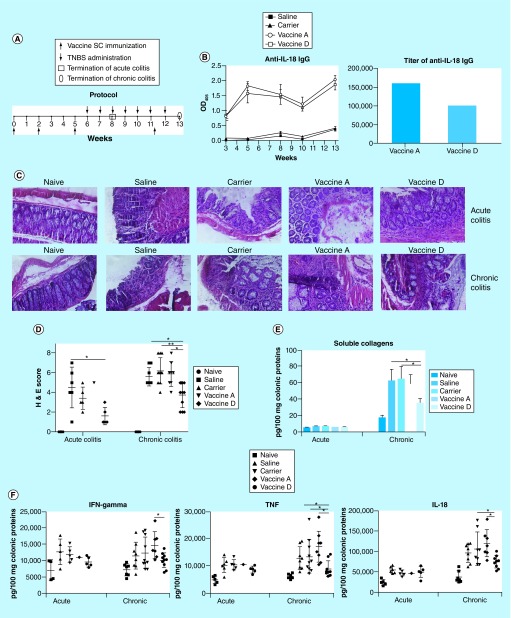
Effects of IL-18 vaccines on intestinal inflammation in mice with TNBS-induced acute and chronic colitis. **(A)** Protocols. n = 6/group for acute colitis, and n = 10/group for chronic colitis. **(B)** IL-18-specific IgG antibody responses induced by vaccines. **(C)** Representative histological inflammation of acute colitis and chronic colitis (original magnification 100×). **(D)** Semi-quantitative analysis of H&E score. **(E)** Soluble collagen productions in colon tissue. **(F)** Cytokine levels in colon tissue. H&E: Haemotoxylin and eosin; TNBS: 2,4,6-trinitrobenzene sulfonic acid.

To evaluate the effects of the IL-18 peptide-based vaccines A and D in the amelioration of TNBS-induced colitis, mice were first immunized with vaccine, carrier or saline three-times to develop high levels of anti-IL-18 antibodies. Following immunization, mice were challenged with TNBS twice to induce acute colitis or seven-times to induce chronic colitis as previously reported (Figure 2A) [19]. Naive mice without TNBS challenges were used as controls. Results showed that serum IL-18-specific IgG levels maintained high levels during entire experimental period in mice receiving either vaccine A or D (Figure 2B). Interestingly, although vaccine A induced slightly higher levels of IL-18-specific IgG than vaccine D, mice immunized with vaccine A did not have any improvement in inflammatory scores, amounts of soluble collagens and IFN-γ, TNF and IL-18 levels in colon tissue in both acute and chronic colitis (Figure 2C–F). In contrast, mice immunized with vaccine D had significant improvements in both acute and chronic intestinal inflammation (Figure 2C–F). In acute and chronic colitis, vaccine D-immunized mice had significant lower H&E score. In chronic colitis, vaccine D-immunized mice had significantly decreased amounts of soluble collagens in the colon tissue when compared with control groups (Figure 2E). The levels of IFN-γ, TNF and IL-18 in colon tissue were also significantly downregulated in vaccine D-immunized mice when compared with controls (Figure 2F). These results indicated that administration of IL-18 vaccine D, not vaccine A, ameliorated TNBS-induced murine colitis.

## Discussion

Studies have revealed that the IL-18 receptor (R) consists of α and β units. On binding of IL-18 to IL-18Rα, which is a low affinity of binding, IL-18Rβ is recruited to form a high affinity receptor and induces signaling pathways [5,22,23]. IL-18Rα is expressed on most cells, while IL-18Rβ is usually expressed on T cells, NK cells and dendritic cells, but not commonly expressed in mesenchymal cells [5]. Although mouse IL-18 structure and binding mode is unknown, through analyzing the position of corresponding peptides of vaccine A and D in human IL-18 (Supplementary Figure 1), it indicates that the corresponding human peptide of vaccine A is located close to site I/II, where is the binding site for IL-18Rα, whiles the corresponding human peptide of vaccine D is located in the site III, where is the binding site for IL-18Rβ [22]. This suggests that IL-18 antibodies induced by IL-18 vaccine A might block the binding of IL-18 to IL-18Rα, while IL-18 antibodies induced by IL-18 vaccine D might block the binding of IL-18Rβ. Studies already indicate that in the absence of IL-18Rβ, binding of IL-18 to IL-18Rα will not induce proinflammatory signals. In the present study, although anti-IL-18 induced by IL-18 vaccine A could partially block IL-18-induced IFN-γ secretion *in vitro*, it did not improve murine colitis *in vivo*. This might be due to that the high concentration of antisera induced by IL-18 vaccine A and relative low concentration of IL-18 were used in the *in vitro* inhibition test, in which anti-IL-18 induced by vaccine A could block the activation of IL-18R signaling pathway. However, *in vivo* it may have low level of anti-IL-18 and high concentration of IL-18 in the local colon tissue, where anti-IL-18 induced by vaccine A could not block the activation of IL-18R signaling pathway. While anti-IL-18 induced by IL-18 vaccine D could partially block IL-18-induced IFN-γ secretion *in vitro* and also improve murine colitis *in vivo*. Therefore, the targeting of different sites of IL-18 by antibodies induced by vaccine A and D, plus the different roles of IL-18Rα and IL-18Rβ chain in inducing signaling pathways, might lead to the different effects of vaccine A and D in the amelioration of murine colitis.

The antibodies induced by IL-18 peptide-based vaccine A and D might have different effects on intestinal epithelial integrity. In gut homeostatic conditions, IL-18 is produced by intestinal epithelial cells (IEC) after NLRP3 or NLRP6 inflammasomes activation in IEC [5,24,25]. IL-18 has a protective role on maintaining both intact intestinal barrier and normal microbiota in the lumen through mucus synthesis by goblet cells or secretion of antimicrobial peptides by IEC [5]. In addition, IL-18 may also limit Th17 differentiation and modulate Treg cell function [26]. In intestinal inflammation, the epithelial barrier is disrupted and bacteria enter massively in the lamina propria, where they induce local macrophages to produce IL-18, which leads to chemokine secretion and leukocyte recruitment from the peripheral blood into the intestinal lamina propria. In the meantime, IL-18 inhibits mucus production by goblet cells and modifies microbiota favoring dysbiosis to exaggerate inflammation [5]. The antibodies induced by IL-18 peptide-based vaccine D might promote the intactness of intestinal barrier through blocking IL-18, which will be explored in the future study.

The present study indicates that peptide D of IL-18 might be a good target to develop antibodies against IL-18, which can block the functions of IL-18. This study also emphasizes that vaccines do not always block or inhibit the activities of their target molecules, but may also have a stimulatory effect. Our group reported that autoantibodies induced by an IL-17 peptide-based vaccine enhanced the biological function of IL-17 both *in vitro* and *in vivo* [17]. Several monoclonal or polyclonal antibodies that enhance the activities of their target proteins were also reported, such as monoclonal or polyclonal antibodies against human insulin-like growth factor I [27] and human immunodeficiency virus type 1 [28,29].

## Conclusion & future perspective

Taken together, IL-18 peptide-based vaccine D induces relatively long-lasting IL-18-specific IgG antibodies, which can block the biological functions of IL-18 *in vitro*. Immunization of IL-18 peptide-based vaccine D is capable of improving TNBS-induced acute and chronic intestinal inflammation. This strategy may provide a potential therapeutic approach for the treatment of Crohn’s disease. Further experiments are clearly needed to address the effects of the vaccines on the binding of IL-18 to IL-18R, intestinal epithelial integrity and infection susceptibility.

**Summary points**

- Six peptides were selected from murine IL-18 based on the occurrence of amino acid residues in experimentally known segmental epitopes and the DNAstar software.
- Two murine IL-18 peptide-based virus-like particle vaccines (vaccines A and D) were successfully developed, which induced relative long-lasting antibodies against murine IL-18.
- Anti-IL-18 induced by IL-18 peptide-based vaccine A and vaccine D could partially block murine IL-18-induced IFN-γ secretion from splenocytes *in vitro*.
- Immunization of mice with IL-18 peptide-based vaccine D, not vaccine A, was capable of improving 2,4,6-trinitrobenzene sulfonic acid-induced acute and chronic murine colitis.
