# Supplementary figures and images for: Sustained suppression of IL-18 by employing a vaccine ameliorates intestinal inflammation in TNBS-induced murine colitis
Source: Future Sci OA. 2019 Jul 30;5(7):FSO405. doi: 10.2144/fsoa-2018-0125 (PMC6695525; doi:10.2144/fsoa-2018-0125)

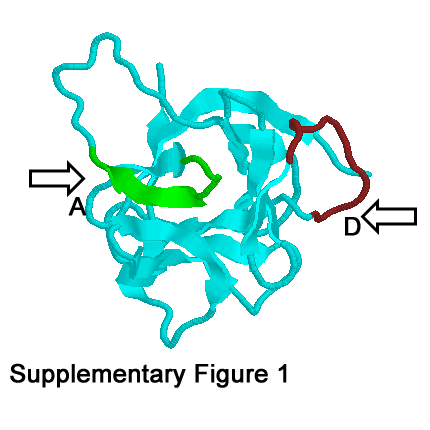

Supplement: Supplementary file 1 [file fsoa-05-405-s1.tif]
